# Assessment of Appetitive Behavior in Honey Bee Dance Followers

**DOI:** 10.3389/fnbeh.2018.00074

**Published:** 2018-04-27

**Authors:** Mariel A. Moauro, M. Sol Balbuena, Walter M. Farina

**Affiliations:** ^1^Laboratorio de Insectos Sociales, Departamento de Biodiversidad y Biología Experimental, Facultad de Ciencias Exactas y Naturales, Universidad de Buenos Aires, Buenos Aires, Argentina; ^2^Instituto de Fisiología, Biología Molecular y Neurociencias (IFIBYNE), CONICET, Universidad de Buenos Aires, Buenos Aires, Argentina

**Keywords:** *Apis mellifera*, waggle dance, gustatory responsiveness, olfactory conditioning, proboscis extension response

## Abstract

Honey bees transfer different informational components of the discovered feeding source to their nestmates during the waggle dance. To decode the multicomponent information of this complex behavior, dance followers have to attend to the most relevant signal elements while filtering out less relevant ones. To achieve that, dance followers should present improved abilities to acquire information compared with those bees not engaged in this behavior. Through proboscis extension response assays, sensory and cognitive abilities were tested in follower and non-follower bees. Individuals were captured within the hive, immediately after following waggle runs or a bit further from the dancer. Both behavioral categories present low and similar spontaneous odor responses (SORs). However, followers exhibit differences in responsiveness to sucrose and odor discrimination: followers showed increased gustatory responsiveness and, after olfactory differential conditioning, better memory retention than non-followers. Thus, the abilities of the dance followers related to appetitive behavior would allow them to improve the acquisition of the dance surrounding information.

## Introduction

The waggle dance is a stereotyped behavior performed by *Apis mellifera* foragers which consists in an eight-shape figure on the vertical comb inside the hive (von Frisch, 1967). This complex behavioral display is considered a multicomponent signal (Grüter and Farina, 2009) which not only attracts nestmates to the dance surrounding but also informs the presence of a profitable food source (von Frisch, 1967; Seeley, 1989). Honey bees can acquire information about the location of the feeding site by following these maneuvers from behind or laterally (Michelsen, 2003; Díaz et al., 2007). The dance followers can also perceive and learn the odors of the collected food during interactions with the dancer (von Frisch, 1967; Farina et al., 2005; Díaz et al., 2007). In this way, both naïve and experienced foragers can acquire information from the waggle dance (Biesmeijer and de Vries, 2001; Biesmeijer and Seeley, 2005).

The honey bee dance takes place in particular comb areas named “dance floor” (Tautz and Lindauer, 1997), located at approximately 4–20 cm from the hive entrance, where dancers and dance followers come into contact (von Frisch, 1967; Seeley, 1995). Dance maneuvers increase the activity of bees in the dancer’s vicinity (von Frisch, 1923; Božič and Valentinčič, 1991; Thom et al., 2007). Thus, in this informational context, the levels of motivation and attention of the bees located in the dance surrounding might be enhanced by the presence of the excited dancers. As a result, follower bees are motivated to start foraging.

The shift from in-hive tasks to foraging involves changes in the responsiveness to external stimuli (Robinson, 1987; Robinson and Page, 1989; Seeley, 1989). Furthermore, bees performing different tasks within the colony also present different response thresholds (Ramírez et al., 2010). In this sense, Katz and Naug (2016) have shown that dancer and follower bees present different sensitiveness according to the individual and colony nutritional states. Creating a mismatch between these nutritional states and using a conditioning assay, they evaluated the proboscis extension response of fed and starved bees. Followers showed to be less sensitive to changes in the colony nutritional condition than dancers. However, incoming foragers can adjust their response to the nutritional status of the colony (Lindauer, 1954; Seeley, 1989; Farina, 2000; De Marco, 2006). Therefore, the presence of individuals responding differentially according to nutritional states would allow a better adjustment of the foraging activity of the entire colony. However, until now, it is unknown how different are the chemosensory and olfactory learning abilities of those bees located in the hive areas where the information related to the incoming resources is transmitted. Bearing this in mind, our aim is to study the sensory and cognitive capacities of bees involved in dance following and unemployed bees located next to the dance floor that did not follow dances at the moment they were captured. For this, we carried out behavioral assays testing sucrose responsiveness, spontaneous response to odors and ability to discriminate odors.

## Materials and Methods

### Study Site and Animals

The experiments were performed during the summer-autumn seasons of 2015 and 2016 at the Experimental Field of the University of Buenos Aires, Argentina (34° 32′S, 58° 26′W). We used four observation hives (two colonies during 2015, henceforth: H1 and H2; and the other two during 2016, henceforth: H3 and H4) that consisted of two frames with brood, a mated queen and about 4000 workers of the European honey bees (*Apis mellifera*) each. The hives were contained between acrylic walls that had a 40 × 25 cm^2^ window covered by a hinged door that allowed access to the colony during the assays. The observation hives were located individually inside a flight chamber (6 m length × 3 m wide × 2 m height), which was closed only during the experiments to prevent interference with other bee colonies. During the rest of the day, the flight chamber was opened for bees to have access to natural food sources.

### General Procedure

A group of foraging workers of each experimental colony was trained to visit an artificial feeder located 6 m from the hive that offered 50% weight/weight (w/w) unscented sucrose solution. Foragers that visited the feeder were marked with acrylic paint (ALBA-Argentina) on their thoraxes to distinguish them from the rest of the nestmates.

Dance maneuvers of bees that returned from the artificial feeder were observed carefully by eye. A successful follower attends 3–7 consecutive waggle-runs before leaving the hive (Judd, 1994). Despite of the short distance to the artificial source, it could be observed waggle dances. Thus, we defined as dance followers those hive bees that were captured directly from the unloading area (dance floor) after following at least three waggle-runs. In addition, we avoided capturing followers that performed oral contact with the dancers in order to avert a gustatory experience which could affect the sensory and cognitive capacities evaluated here (see sections below; Farina et al., 2007; Martinez and Farina, 2008). Unemployed hive bees that were not observed to be involved in dance following (henceforth: non-follower bees) were caught at a distance of 10–20 cm from the dancers, depending on the size of the dancing area (considering the approximate followed waggle dance radius is about 1.5 cm), and were used to compare their spontaneous odor response (SOR), gustatory responsiveness and odor discrimination with the dance follower’s responses. Bees that performed cell cleaning, food and wax processing or absolute repose were not captured. It is worth to note that bees of both categories were caught from combs without brood or food reserves.

The captured bees were anesthetized in the freezer (−18°C) for no more than 2 min and harnessed in small metal tubes that restrained body movement but allowed free movement of antennae and mouthparts (Frings, 1944; Bitterman et al., 1983; Matsumoto et al., 2012). After waking, bees were offered a drop of water to drink if they would be tested for their gustatory responsiveness or 30% w/w sucrose solution if they would be submitted to the differential conditioning protocol and then, housed in an incubator (30°C, 55% RH and darkness) for 90 min before assessing their response.

### Spontaneous Odor Response

The proboscis extension reflex (PER) is a reliable indicator for studying response to odors or sugared rewards, such as those found in nectar naturally (Frings, 1944; Page et al., 1998). Even though an odor is considered a neutral stimulus and usually the proboscis is not released by itself, a novice bee can still show a spontaneous response towards certain odors in the laboratory context (Guerrieri et al., 2005). For this procedure, two pure odors commonly present in floral fragrances (Knudsen et al., 1993; Raguso and Pichersky, 1999; Nouvian et al., 2015), Linalool (LIO) and Phenylacetaldehyde (PHE; Sigma-Aldrich, Steinheim, Germany), were used to test the SOR of bees from H1 and H2. A device that delivered a continuous airflow was used for the odorant application. Individually harnessed bees were exposed to a constant clean airstream (2.5 ml s^−1^) delivered 2 cm away from their heads. A filter paper (30 × 3 mm) was impregnated with 4 μl of the pure odorant and placed inside a syringe. Each odor was delivered during 6 s when the airflow was redirected to pass through the syringe by means of an electric valve. A SOR was measured when the bee fully extended its proboscis towards any of the two odors, during odor delivery (Farina et al., 2005). Bees that responded to the mechanical air stimulus (6 s of clean airflow before and 3 s after odor presentation) were discarded, as well as bees that did not respond to 50% w/w sucrose solution after the gustatory response assay. The elapsed time between the first and the second odor presentation was 15 min, and the order was alternated from bee to bee.

### Gustatory Responsiveness

PER was also used to evaluate gustatory responsiveness, by determining a gustatory response score (GRS) per experimental bee (Page et al., 1998). It was tested on bees from H1 and H2 after going through SOR assay. At the beginning of the assay, water was offered in order to avoid confounding thirst effects. Bees were assayed by presenting sucrose solutions of increasing concentration (0.1, 0.3, 1, 3, 10, 30 and 50% w/w; Page et al., 1998). Usually, this protocol only tests the response to sucrose concentrations from 0.1% to 30% w/w; however, we added the 50% w/w solution in order to include those bees with high sucrose response thresholds. Between each concentration of sucrose solution, all bees were tested for their response to water. This was done to avoid potential effects of repeated sucrose stimulation that could lead to increased sensitization or habituation. The inter-stimulus interval between water and sucrose solution was an average of 3 min. At the end of the procedure, a GRS was obtained for each bee. This score was based on the times each bee responded to the different sucrose concentrations (Scheiner et al., 2001; Pankiw et al., 2004). The response was arbitrarily quantified with scores from *one* to *seven*, where one (1) represented a bee that only responded to the highest sucrose concentration (50% w/w), while a score of seven (7) represented an individual that responded to all concentrations tested. If a bee failed to respond to a concentration of sucrose in the middle of a response series (e.g., responded to 0.1, 0.3, 3 and 10% w/w, but did not respond to 1%), this response was considered to be an error and the bee was deemed to have responded to that concentration as well. A bee that did not respond to more than one of the sucrose concentration in the middle of a response series was excluded from the analyses (Mc Cabe et al., 2007; Martinez and Farina, 2008). The same happened for bees that did not respond to the 50% w/w concentration (positive control, score = 1) offered because we cannot assure if they were able to sense and respond. In addition, those bees that responded to all sucrose concentrations but to all water presentations, too, were excluded from analyses as they appeared not to be able to discriminate between sucrose solution and water, were too starved or just thirsty.

### Differential PER Conditioning

Two additional hives, H3 and H4, were exposed to 1.5 ml of a pure odor for at least 3 days before performing PER conditioning (H3: PHE, H4: LIO). The scent was provided by two small Petri dishes placed at the bottom of the hive containing a filter paper (3 cm diameter) soaked with 0.75 ml of the pure scent. The Petri’s top lid was perforated to prevent bees touching the compound while volatiles could be dispelled. The volatile exposed in the hive had the purpose that bees associate it with a non-appetitive hive context (Farina et al., 2005; Díaz et al., 2007).

The bees captured from both colonies were subjected to a differential PER conditioning to analyze olfactory discrimination to two pure odors during training procedure (Bitterman et al., 1983): the rewarded odor (rewarded conditioning stimulus, CS+) was paired with 50% w/w sucrose solution (unconditioned stimulus, US), and the non-rewarded odor was presented alone (non-rewarded conditioned stimulus, CS−) and was the same used in the hive as the pre-exposed odor. The same device described in SOR section was used for this assay. The volatile compound was delivered through a secondary air stream (2.5 ml s^−1^) injected into the main airflow during the delivery of the odor. The bees were exposed to these stimuli five times each in a pseudo-randomized order (CS−, CS+, CS+, CS−, CS−, CS+, CS−, CS+, CS+, CS−). For each colony, differential PER conditioning was carried out using a pre-exposed odor as CS− and a non-exposed odor as CS+ (H3: CS− = PHE, CS+ = LIO; H4: CS− = LIO, CS+ = PHE). The inter-trial interval lasted 10–15 min between the CS presentations, depending on the number of individuals tested at one time (usually, *n* = 20–40). Only those bees that showed an unconditioned response (the reflexive extension of proboscis after applying a 50% w/w sucrose solution to the antenna, UR) and that did not respond to the mechanical airflow stimulus were used for the test. During the experiment in the per setup, a fan extracted the released odors to avoid contamination. Each learning trial lasted 40 s and we presented the CS for 6 s. Reinforcement (50% w/w sucrose) was delivered for 3 s after the onset of the CS+. Those bees that showed a spontaneous response to the odor in the first trial were discarded from the experiment. To evaluate whether the bees had formed a medium-term memory after they had all gone through the learning assay, bees stayed harnessed for 15 min and were then subjected to a non-rewarded presentation of both training odors (testing phase).

### Statistics

The effects of factors on all variables were assessed by means of Generalized Linear Models (GLM) or Generalized Linear Mixed Models (GLMM), depending on the type of factors included in the models. Models were fitted in R program (R Development Core Team, 2011) using the *glm* function for the former case and the *glmer* function of the lme4 package (Bates et al., 2015) for the latter, in which fixed and random effects are specified via the model formula. We used the MuMIn package which contains functions to streamline information-theoretic model selection and carry out model averaging based on the information criteria (Burnham and Anderson, 2002). The *dredge* function was used to perform the automated model selection with subsets of the global model; the set of models was generated with all possible combinations of the fixed factors. *AICc*, which is a second-order information criterion, was used to rank the models and to obtain model weights (Akaike, 1973). Unless the Akaike weight for the best model was high enough (*w*_k_ ≥ 0.8, personal criterion), we could not consider that the predictors not selected were unimportant; so, in the cases in which the *w*_k_ < 0.8, we performed multimodel inference using the *model.avg* function which calculates model averaged parameters with standard errors and confidence intervals to evaluate the significances.

The effect of the dance following behavior on SOR was assessed by means of a GLM with binomial error structure. Gustatory responsiveness was estimated through the GRS, which is a sum of the unconditioned responses to the sugar solutions presented in the procedure. Values include 1 through 7. The effect of the following or non-following behavior on GRS was assessed by means of a GLM with Poisson error structure. In both cases, the initial model included *behavior* and *hive* as fixed factors, considering only additive effects. *Hive* was considered as a fixed factor because it had only two levels (Zuur et al., 2007). In the particular case of GRS, in which significant difference between the hives was found, we analyzed *behavior* factor in each hive separately by constructing data subsets.

The effect of behavior on olfactory discrimination was assessed by means of a GLMM with binomial error structure. The initial model to analyze the acquisition phase included *behavior*, *trial* and *hive* as fixed factors, contemplating only additive effects, and *bee* as a random factor. In the testing phase, none of the bees extended its proboscis towards the CS−. Therefore, the effect of behavior was only studied on conditioned response towards the CS+ and assessed by means of a GLM which included *behavior* and *hive* as fixed factors, considering additive effects.

## Results

### Spontaneous Odor Response

**Figure 1:**
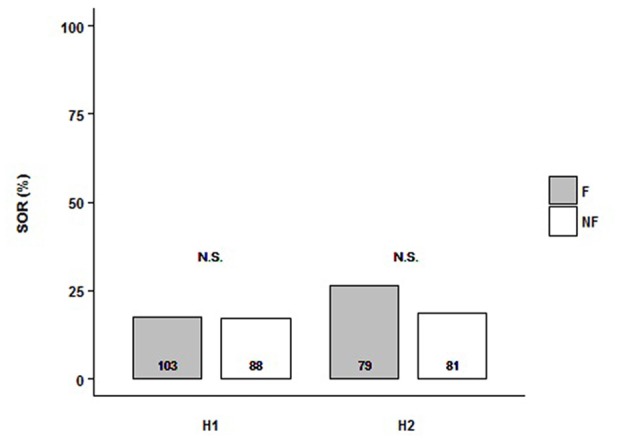
Follower and non-follower dancing honey bees present similar probability of spontaneous odor response (SOR). Percentage of bees, followers (F, gray bars) and non-followers (NF, white bars) that extended their proboscises towards any of the two odors, Linalool (LIO) and Phenylacetaldehyde (PHE). Bees from Hive 1 (H1) and Hive 2 (H2) were tested. “N.S.” indicates no statistical differences (*behavior*, as a factor, is not included in the final model; see “Results” section for details). The number of bees tested is shown inside the bars.

We tested the SOR of follower and non-follower bees from H1 and H2. Each experimental bee was exposed to a single presentation of two odor stimuli in the per setup: LIO and PHE. Because we were interested in the response to the odors independently of their identity, we analyzed the SOR towards any of them. Then, we grouped the odors since there was no interaction with the behavior fixed factor (in all cases, there was a bit higher response to PHE; data not shown). For both hives, we found that, independently of the behavioral category analyzed (followers vs. non-followers), bees showed low SOR levels (ca. 25%) and there was no difference between them (H1: *N*_F_ = 103, *N*_NF_ = 88; H2: *N*_F_ = 79, *N*_NF_ = 81). Indeed, the minimal model did not include the behavioral factor (Figure 1, Supplementary Table S1; SOR_H1+H2_~1). Thus, both followers and non-followers did not show odor preference. Although there was no significant difference between colonies, Figure 1 shows their result separately because, analyzing GRS of the same bees (see below), *hive* factor was included in the minimal model.

### Gustatory Response Scores

**Figure 2:**
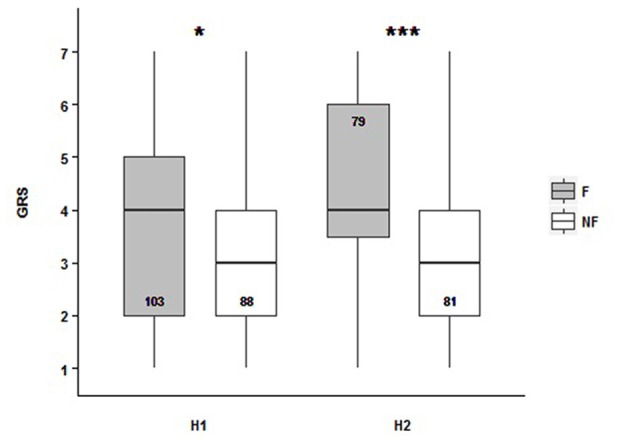
Follower and non-follower dancing honey bees present different gustatory responsiveness. Gustatory response score (GRS) for followers (F, gray bars) and non-followers (NF, white bars) from Hive 1 (H1) and Hive 2 (H2). Medians (black line), quartiles as vertical boxes, and ranges with bars are shown. Median values: for followers, 4, and for non-followers, 3. Asterisks indicate statistical differences (^*^p < 0.05; ^***^p < 0.001; see “Results” section for details). The number of bees tested is shown inside the boxes.

After SOR, our aim was to study if gustatory responsiveness was affected by the dance context. We evaluated the sucrose response thresholds of bees from H1 and H2 under the PER paradigm. The GRS was defined as the sum of positive responses throughout the presentation of increasing concentration of the sucrose solutions (Page et al., 1998). Our results show that the dance followers had a higher GRS than non-follower bees in both experimental colonies [median values: 4 for followers (*N*_H1_ = 103, *N*_H2_ = 88), and 3 for non-followers (*N*_H1_ = 79, *N*_H2_ = 81)]. It means that follower bees present higher sucrose responsiveness than non-followers: followers respond to lower sucrose concentrations (Figure 2, Supplementary Table S2; GRS_H1_~ Behavior, *Z* = −2.462, *p* = 0.0138; GRS_H2_~ Behavior, *Z* = −3.899, *p* = 9.65e^−05^). Hence, the gustatory responsiveness seems to be dependent on the behavioral category which bees belonged to. Even though we found significant difference between colonies, it is important to highlight that the tendency for both of them, related to *behavior* factor, was the same, which means there was no interaction between the analyzed factors.

### Odor Discrimination in Classical PER Conditioning

**Figure 3:**
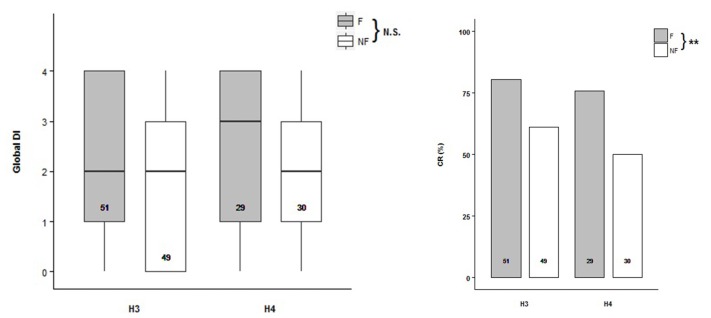
Olfactory learning abilities of the follower and non-follower dancing honey bees.** (A)** Global Discrimination Indexes (Global DI) during acquisition, and **(B)** percentage of bees that extended their proboscis towards the rewarded conditioned stimulus (Conditioned Response, CR) during the testing phase performed 15 min after acquisition. Follower (F, gray bars) and non-follower bees (NF, white bars) from hive 3 (H3) and hive 4 (H4) were tested. “N.S.” indicates no significant differences while asterisks indicate statistical differences (^**^p < 0.01; see “Results” section for details). The number of bees tested is shown inside the boxes and the bars.

Odor discrimination were tested in bees from scent pre-exposed hives H3 and H4 (H3: *N*_F_ = 51, *N*_NF_ = 49; H4: *N*_F_ = 29, *N*_NF_ = 30) under PER conditioning procedure. Global Discrimination Index (DI) was defined for each bee as the number of trial pairs the bee succeeded in discriminating between the two odors, in other words, if it extended its proboscis towards the CS+ but not to the CS− (Mengoni Goñalons et al., 2016). No difference was found during the acquisition between the two behavioral groups of bees (Figure 3A, Supplementary Table S3; ACQ_H3+H4_~Trial+1|Bee). However, the acquisition level reached by the follower bees at the last trial was between 10% and 15% higher than by the non-followers, in both hives (H3: *F* = 73%, *NF* = 63%; H4: *F* = 72%, *NF* = 57%). During the testing phase, the follower bees exhibited a higher Conditioning Response (CR) to CS+ than the non-follower ones (Figure 3B, Supplementary Table S4; TEST_H3+H4_~Behavior, *Z* = −2.724, *p* = 0.00646). Therefore, followers showed better memory retention than the non-follower when they were evaluated 15 min after the olfactory conditioning.

## Discussion

Through PER assays, we evaluated olfactory and gustatory responsiveness besides the ability to discriminate odors through classical conditioning in honey bee workers with different probabilities to be recruited as foragers. A brief time after capturing both dance follower and non-follower bees showed similar SOR levels but significant differences in the sucrose responsiveness and odor memory retention. Specifically, dance followers presented higher gustatory responsiveness (higher GRS levels) and better memory retention after olfactory conditioning than non-followers.

We did not take into account the age of the experimental bees in this study. Previous reports suggest that age seems not to be relevant within this social context. For instance, individualized bees that followed dances showed a wide age range (i.e., from 9 to 32 days old; Balbuena et al., 2012). Likewise, a recent study evaluated the sucrose responsiveness of hive bees captured from the dance floor or delivery area within an age interval of 2–15 days old (Mengoni Goñalons et al., 2016). No age-dependent relationship was found for the sucrose responsiveness measured at the per setup in that study. In this sense, the gustative responsiveness of honey bees may be affected by the informational context where individuals were caught from (i.e., dance floor) more than by their age. Indeed, Martinez and Farina (2008) showed that hive bees captured after receiving food from a donor bee presented different sucrose responsiveness according to the food quality received during the oral contact.

In the present study, bees captured in the dance context (following dances but did not interact orally with a dancer) showed higher gustatory responsiveness compared with those bees captured at a distance of 10–20 cm from the dancer. High GRS values correlate positively with improved performances during olfactory conditioning as it was previously reported (Scheiner et al., 2004; Mengoni Goñalons and Farina, 2015). Consistent with this evidence, we found that dance followers showed improved levels of memory retention after an olfactory PER conditioning. However, when sucrose responsiveness correlates with memory retention, also correlates with learning performance (Scheiner et al., 2001, 2004; Mengoni Goñalons and Farina, 2015). Here, we only found a correlation between memory retention and gustatory responsiveness. Those previous studies used absolute conditioning procedures to test it. In this study, differential conditioning has been used. This protocol evaluates the ability to associate an odor to a reward, but also the capacity to distinguish it from another which is not linked to an unconditioned appetitive stimulus. Here, our unrewarded odor, CS−, is the same odor used that we used as the hive odor and it would represent a nonappetitive context for the experimental subjects. Thus, the differences in response to rewarded and unrewarded odors would be bigger for those bees that are more motivated to acquire appetitive information. Although it was not significant, this tendency can be observed at the end of the learning performance for dance followers and clearly visualized while the trained odors were tested 15 min later. It is worth mentioning that we only evaluated memory retention at a medium-term scale. It is expected that highly motivated individuals not only learn faster but also recall longer, an issue which was not cover in the present study.

Changes in the motivation and attention levels of the dance followers would turn out to be more sensitive to any other environmental stimuli, a fact that might facilitate the decoding of spatial information transmitted as well as the acquisition of incidental cues such as floral odors carried by the waggle dancer. The role of the early odor-rewarded experiences acquired in the beehive as a stimulus that facilitates the decoding of waggle dance information at elder ages has been suggested (Balbuena et al., 2012). In that study, honey bees preferred to follow dancers scented with an early exposed and rewarded odorant, and even they were recruited to the feeding site scented with the early experienced odors more successful. As the presence of reward affects physiological states in honey bees within a short-term period (Hammer, 1997), the most vigorous dances, which indicate the presence of a highly profitable food source, might represent appetitive stimuli that facilitate a prompt acquisition of information within the dance context.

This study shows a correlation between sensory and cognitive performances and behavioral category based on the dance context. Nevertheless, it does not show if there is a causal relationship. To do that, the life history of individual bees should be considered to determine whether bees with a low gustatory responsiveness tend to follow dances or even whether the sucrose responsiveness changes after dance following. Our results are a first approach to understand abilities of the dance surrounding bees, but this issue requires further analysis.

## Author Contributions

MAM, MSB and WMF contributed to the conception and design of the study and contributed to the drafting and revision of the manuscript and approved the final version. MAM and MSB conducted the experiments. MAM analyzed the data.

## Conflict of Interest Statement

The authors declare that the research was conducted in the absence of any commercial or financial relationships that could be construed as a potential conflict of interest.

## Supplementary Material

The Supplementary Material for this article can be found online at: https://www.frontiersin.org/articles/10.3389/fnbeh.2018. 00074/full#supplementary-material
