# Supplementary material for: Assessment of Appetitive Behavior in Honey Bee Dance Followers
Source: Front Behav Neurosci. 2018 Apr 27;12:74. doi: 10.3389/fnbeh.2018.00074 (PMC5934941; doi:10.3389/fnbeh.2018.00074)
Supplement: Supplementary file 1 [file Data_Sheet_1.pdf]

Supplementary Material

Assessment of appetitive behavior in honey bee dance followers

Mariel A. Moauro†, M. Sol Balbuena†, Walter M. Farina[*¶]

† These authors have contributed equally to this work.

* Corresponding author: e-mail: walter@fbmc.fcen.uba.ar

Table S1. Set of generalized linear models that can explain variability in the honey bees´ spontaneous odor response

Hive	Model	AICc	Δ_i	w_i
H1+H2	SOR~ 1	349.9	0.00	0.367
	SOR ~ Hive	350.5	0.52	0.282
	SOR ~ Behavior	351.2	1.27	0.194
	SOR ~ Hive+Behavior	351.6	1.70	0.157

MMI

Hive		CI2.5%	CI97.5%
H1+H2	(Intercept)	-1.9411480	-1.1060866
	Hive2	-0.1964185	2.012371
	Followers	-0.2916715	0.7745214

AICc is a second-order AIC, necessary for small samples. Δ_i is AIC differences, relative to the smallest AIC value in the set of R models. Formally, $\Delta_i = AIC_i - AIC_{min}$ is an estimation of distance between the best model and the *i*th model. Akaike weights, denoted by w_i, are the relative likelihood of the model. There are normalized to sum to 1 and interpreted as probabilities. None of the models reached a weight of 0.8, so we applied a *multimodel inference* (MMI), to predict the levels significance. All confidence intervals (CI) include 0, so none of the factors resulted to be significant to construct the minimal model.

Table S2. Set of generalized linear models that could explain the variability in the honey bees´ gustatory response score

Hive	Model	AICc	Δ_i	w_i
H1+H2	GRS ~ Behavior+Hive	1369.9	0.00	0.788
	GRS ~ Behavior	1372.5	2.62	0.212
	GRS ~ Hive	1388.2	18.29	0.000
	GRS ~ 1	1390.0	20.09	0.000

MMI

Hive		CI2.5%	CI97.5%
H1+H2	(Intercept)	1.01144619	1.2292821
	Hive2	0.01075785	0.2299772
	Followers	0.14079860	0.3628541

SUBSET

Hive	Model	AICc	Δ_i	w_i
H1	GRS ~ Behavior	751	0.00	0.885
	GRS ~ 1	755	4.08	0.115
H2	GRS ~ Behavior	619.8	0.00	0.999
	GRS ~ 1	633.2	13.32	0.001

AICc is a second-order AIC, necessary for small samples. Δ_i is AICc differences, relative to the smallest AIC value in the set of R models. Formally, $\Delta_i = AIC_i - AIC_{min}$ is an estimation of distance between the best model and the *i*th model. Akaike weights, denoted by w_i, are the relative likelihood of the model. There are normalized to sum to 1 and interpreted as probabilities. None of the models reached a weight of 0.8, so we applied a *multimodel inference* (MMI) to predict the levels significance. The confidence intervals (CI) that did not include 0 correspond to the *hive* and *behavior* factors. As there was significant difference between hives, we decided to construct a data subset of each hive to evaluate *behavior* factor.

Table S3. Set of generalized linear models that could explain the variability in the honey bees´ acquisition during differential conditioning

Hive	Model	AICc	Δ_i	w_i
H3+H4	ACQ ~ Behavior+Trial+1|bee	690.6	0.00	0.371
	ACQ ~ Trial+1|bee	690.7	0.12	0.350
	ACQ ~ Behavior+Trial+Hive+1|bee	692.5	1.89	0.144
	ACQ ~ Trial+Hive+1|bee	692.6	2.02	0.135
	ACQ ~ Behavior+1|bee	769.3	78.71	0.000
	ACQ ~ 1|bee	769.5	78.91	0.000
	ACQ ~ Behavior+Hive+1|bee	771.2	80.59	0.000
	ACQ ~ Hive+1|bee	771.4	80.79	0.000

MMI

Hive		CI2.5%	CI97.5%
H3+H4	(Intercept)	-2.66846560	-0.90798960
	Trial3	1.21580930	2.63320780
	Trial4	1.70324160	3.19582560
	Trial5	2.20978350	3.80418340
	Followers	-0.24850660	1.70056700
	Hive4	-0.81230430	1.20408530

AICc is a second-order AIC, necessary for small samples. Δ_i is AICc differences, relative to the smallest AIC value in the set of R models. Formally, $\Delta_i = AIC_i - AIC_{min}$ is an estimation of distance between the best model and the *i*th model. Akaike weights, denoted by w_i, are the relative likelihood of the model. There are normalized to sum to 1 and interpreted as probabilities. None of the models reached a weight of 0.8, so we applied a *multimodel inference* (MMI) to predict the levels significance. The confidence intervals (CI) that did not include 0 correspond to the *trial* factor. There is significant difference between all of its levels.

Table S4. Set of generalized linear models that could explain the variability in the honey bees´ odor response after differential conditioning

Hive	Model	AICc	Δ_i	weight
H3+H4	TEST ~ Behavior	197.4	0.00	0.613
	TEST ~ Behavior+Hive	198.6	1.24	0.330
	TEST ~ 1	203.0	5.66	0.036
	TEST ~ Hive	204.2	6.82	0.020

MMI

Hive		CI2.5%	CI97.5%
H3+H4	(Intercept)	-0.17690320	0.87520660
	Followers	0.26200110	1.65058500
	Hive4	-1.02702480	0.37491670

AICc is a second-order AIC, necessary for small samples. Δ_i is AICc differences, relative to the smallest AICc value in the set of R models. Formally, $\Delta_i = AIC_i - AIC_{min}$ is an estimation of distance between the best model and the *i*th model. Akaike weights, denoted by w_i, are the relative likelihood of the model. There are normalized to sum to 1 and interpreted as probabilities. None of the models reached a weight of 0.8, so we applied a *multimodel inference* (MMI) to predict the levels significance. The only confidence interval (CI) that did not include 0 corresponds to the *behavior* factor. It means that there is significant difference between its levels (follower, non-follower).
